# Presence of atypical porcine pestivirus (APPV) genomes in newborn piglets correlates with congenital tremor

**DOI:** 10.1038/srep27735

**Published:** 2016-06-13

**Authors:** Alexander Postel, Florian Hansmann, Christine Baechlein, Nicole Fischer, Malik Alawi, Adam Grundhoff, Sarah Derking, Jörg Tenhündfeld, Vanessa Maria Pfankuche, Vanessa Herder, Wolfgang Baumgärtner, Michael Wendt, Paul Becher

**Affiliations:** 1 University of Veterinary Medicine, Department of Infectious Diseases, Institute of Virology, EU and OIE Reference Laboratory for Classical Swine Fever, Hannover, 30559, Germany; 2 University of Veterinary Medicine, Department of Infectious Diseases, Institute of Virology, Hannover, 30559, Germany; 3 University of Veterinary Medicine, Department of Pathology, Hannover, 30559, Germany; 4 University of Veterinary Medicine, Center for Systems Neuroscience, Hannover, 30559, Germany; 5 University Medical Center Hamburg- Eppendorf, Institute for Medical Microbiology, Virology and Hygiene, Hamburg, 20246, Germany; 6 Leibniz Institute for Experimental Virology, Heinrich Pette Institute, Hamburg, 20251, Germany; 7 Veterinary practice Vetland^®^ Dr. Tenhündfeld & Kollegen, Vreden, 48691, Germany; 8 University of Veterinary Medicine, Clinic for Swine, Small Ruminants, Forensic Medicine and Ambulatory Service, Hannover, 30173, Germany

## Abstract

Pestiviruses are highly variable RNA viruses belonging to the continuously growing family *Flaviviridae*. A genetically very distinct pestivirus was recently discovered in the USA, designated atypical porcine pestivirus (APPV). Here, a screening of 369 sera from apparently healthy adult pigs demonstrated the existence of APPV in Germany with an estimated individual prevalence of 2.4% and ~10% at farm level. Additionally, APPV genomes were detected in newborn piglets affected by congenital tremor (CT), but genomes were absent in unaffected piglets. High loads of genomes were identified in glandular epithelial cells, follicular centers of lymphoid organs, the inner granular cell layer of the cerebellum, as well as in the trigeminal and spinal ganglia. Retrospective analysis of cerebellum samples from 2007 demonstrated that APPV can be found in piglets with CT of unsolved aetiology. Determination of the first European APPV complete polyprotein coding sequence revealed 88.2% nucleotide identity to the APPV sequence from the USA. APPV sequences derived from different regions in Germany demonstrated to be highly variable. Taken together, the results of this study strongly suggest that the presence of APPV genomes in newborn piglets correlates with CT, while no association with clinical disease could be observed in viremic adult pigs.

Pestiviruses are enveloped highly variable RNA viruses with a genome of about 12.3 kb belonging to the family *Flaviviridae*^1^. Besides the classical pestiviruses bovine viral diarrhea virus-1 (BVDV-1), BVDV-2, border disease virus (BDV) and classical swine fever virus (CSFV), a growing number of additional tentative pestivirus species was discovered in the last few years^2^,^3^,^4^. Until recent discovery of very distantly related pestivirus sequences in bats and rats, it was commonly accepted that pestivirus infections are limited to ungulate hosts^2^,^3^. Infections with the classical pestivirus species are worldwide of utmost socioeconomic relevance. In consequence, many countries have implemented compulsory eradication programs for bovine viral diarrhea (BVD) and classical swine fever (CSF), the latter is also reportable to the World Organization for Animal Health (OIE). Besides dramatic clinical signs and high mortality rates particularly observed in acute infections with highly virulent CSFV strains, fetopathogenicity is a common feature of intrauterine pestivirus infections and has significant consequences for productivity of animal breeding^5^.

Recently a novel, genetically very distinct pestivirus, tentatively designated “atypical porcine pestivirus” (APPV), was discovered in pigs from the USA by high throughput sequencing^6^. So far, the clinical relevance of APPV infections remained elusive. In this study, we report the detection of APPV genomes in serum of apparently healthy pigs from two different Federal states in Germany. In addition, APPV genomes were identified in different tissues including cerebellum and peripheral nerves of piglets with congenital tremor (CT, *Myoclonia congenita*) but not in healthy piglets from the same herd providing evidence for a so far unknown association with CT in newborn piglets.

## Results

### Identification of APPV in healthy adult pigs and newborn piglets with CT

Two different SYBR-Green based APPV RT-PCRs targeting the NS3 and NS4B encoding regions of APPV were developed based on the only available APPV sequence from the USA and taking into account other atypical pestivirus sequences from rat and bat. A total number of 369 serum samples from clinically unsuspicious sows and fattening pigs originating from South Germany (20 farms, 200 sera) and North Germany (9 farms, 169 sera) were screened with these RT-PCRs. Both assays identified APPV genomes in three sows (two herds) from Bavaria and six finishing pigs (one herd) from Lower Saxony resulting in an individual genome prevalence of 2.4% and a prevalence of ~10% at farm level.

Several farms located in the western part of Germany experienced cases of CT becoming evident by unintended shivering of newborn piglets, but no clinical signs in the sows or other adult pigs were observed. The etiology of this clinical syndrome is elusive, but an infectious etiology was suspected due to the regional accumulation of cases and epidemiological links. To exclude CSFV infection, eight piglets (six affected and two unaffected animals) from three affected litters were sacrificed for pathological and virological investigations. As no viral genomes of CSFV and other established pestivirus species were detectable by generic Pan-Pestivirus RT-PCR, the two SYBR-Green based APPV-specific PCRs were applied. All of the six clinically affected piglets showed APPV genomes in serum, cerebrospinal fluid and pooled central nervous system samples (comprising cerebrum, cerebellum, and spinal cord), while both piglets without tremor were tested negative (Table 1). Sera (n = 23) obtained from sows of the affected farm with and without affected litters were negative for APPV genomes.

### Epidemiology

Outbreaks of CT were observed in several farms located in Western Germany (North Rhine-Westphalia) in 2015. With the exception of one litter (out of 50 affected litters in total) only litters of newly introduced gilts from the same multiplier herd were affected. Clinical cases of CT appeared in the investigated sow herd (720 “DanBred” hybrid sows) since August 2015 after changing the source of breeding stock by placing of new gilts. On average 30–40% piglets of the affected litters showed typical symptoms of CT. Most of them recovered within two to three weeks, total piglet mortality in the herds remained unchanged.

### Post mortem examination

**Figure 1:**
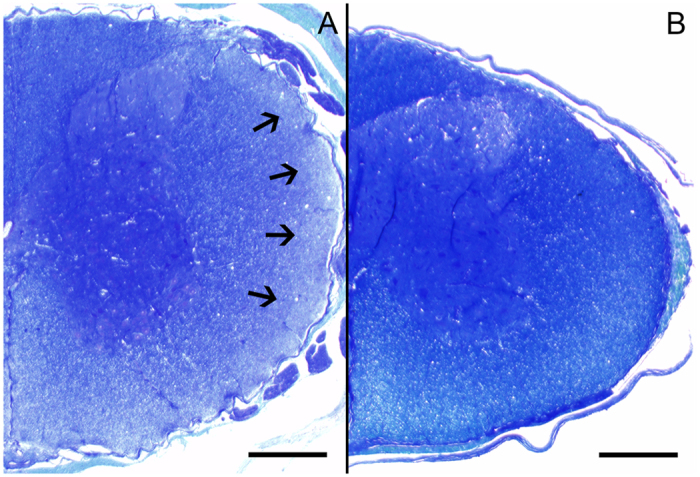
Histochemical visualization of myelin using Luxol fast blue cresyl fast violet staining in the spinal cord of two days old piglets. Congenital tremor affected piglet (no. 52) showed a mildly reduced myelin staining intensity accentuated in the lateral white matter (**A**, indicated by arrows) compared to an unaffected piglet (piglet no. 53) with regular myelination (**B**). Scale bars = 500 μm.

Necropsies were performed on six diseased and two clinically unaffected piglets (two days of age). Gross lesions were restricted to a local facial dermatitis in seven out of eight piglets. Histologically, a mild suppurative omphalitis was detected in four out of eight piglets while central and peripheral nervous system as well as skeletal muscle were without significant findings. Luxol fast blue staining revealed a mild reduced staining intensity accentuated in the lateral white matter of the spinal cord in four out of six diseased animals while the two age-matched, clinically unaffected animals showed a regular myelination (Fig. 1).

### Tissue tropism of APPV

**Figure 2:**
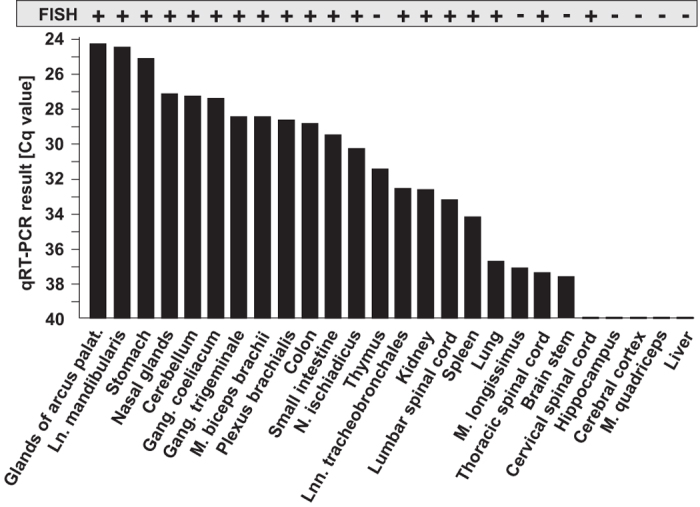
Tissue tropism of atypical porcine pestivirus (APPV). Detection of APPV genomes in different tissues of a two day old piglet with congenital tremor by fluorescent *in-situ* hybridization (FISH) and quantitative reverse transcription polymerase chain reaction (qRT-PCR). Organ distribution of APPV genome as detected by FISH is shown as present (+) or absent (−). Furthermore, for each organ the respective quantification cycle (Cq) values are given. Ln = Lymphonodus, Gang = Ganglion, M = Musculus, N = Nervus, Lnn = Lymphonodi.

**Figure 3:**
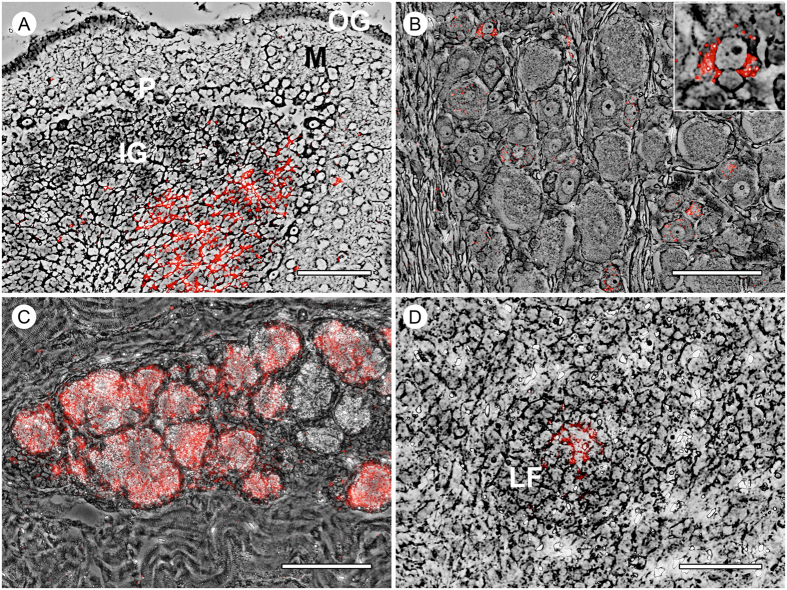
Fluorescent *in-situ* hybridization showing the organ tropism of atypical porcine pestivirus (APPV) in a two day old piglet with congenital tremor. APPV genome (red) was detected in the inner granular cell layer of the cerebellum (**A**), cytoplasm of spinal ganglia neurons (**B**), glandular epithelial cells of the *Arcus palatoglossus* (**C**) and in follicular centers of the mandibular lymph node (**D**). Insert in B shows a higher magnification of an APPV positive neuron. OG = outer granular cell layer, P = Purkinje cell layer, IG = inner granular cell layer of cerebellum; M = molecular layer; LF = lymphoid follicle; scale bars in A–C = 100 μm and D = 50 μm.

26 organ samples from one affected piglet (no. 59) were analyzed by quantitative RT-PCR (qRT-PCR) with a specific TaqMan probe to determine the tissue tropism of APPV (Fig. 2). Highest genome loads were found in glands of the *Arcus palatoglossus* and the *Lymphonodus mandibularis* (quantification cycles, Cq values: 24.3 and 24.4). Other tissues that typically contain high genome loads in case of CSFV infection, like kidney and spleen, gave a much weaker signal corresponding to approximately 1000 fold less APPV genome equivalents. Cerebellum, trigeminal and spinal ganglia revealed to contain high amounts of APPV genomes, but also peripheral nerves were tested positive. In addition, all cerebellar samples obtained from diseased piglets contained high loads of APPV genomes, while both clinically unaffected piglets were APPV negative (Table 1). Fluorescent *in-situ* hybridization (FISH) substantiated the results of the TaqMan qRT-PCR with exception of a positive PCR result obtained for the thymus that could not be confirmed by *in situ* hybridisation (Fig. 2). Three tissues containing very low genome loads (*M. longissimus*, brain stem, cervical spinal cord) gave inconsistent results probably due to the detection limits of both assays and uneven distribution of APPV positive cells in the tissues. In the central nervous system, virus genome was located in the inner granular cell layer of the cerebellum (Fig. 3A) as well as in spinal (Fig. 3B) and trigeminal ganglia. Furthermore, glandular epithelial cells in the *Arcus palatoglossus* (Fig. 3C) and Brunner’s glands (duodenum) as well as lymphoid organs showed a strong positive signal which was most prominent in the follicular centres (Fig. 3D).

### Evidence for a common association of APPV genomes in cerebellum samples and CT

Comparable to the recent outbreak, several sow farms were affected by CT in 2007, after placing of new gilts from the same multiplier herd (“JSR Hybrid” sows). For retrospective analyses, formalin-fixed and paraffin-embedded cerebellum samples archived from this earlier outbreak were analyzed by real-time PCR and *in situ* hybridization. Two out of eleven cerebellar samples showed a positive result in both APPV PCRs (NS3 and NS4B genomic regions). FISH of the cerebellum revealed a strong APPV specific signal mainly located in the inner granular layer of the cerebellum in both diseased piglets (data not shown).

### Molecular characterization of APPV genomes

**Figure 4:**
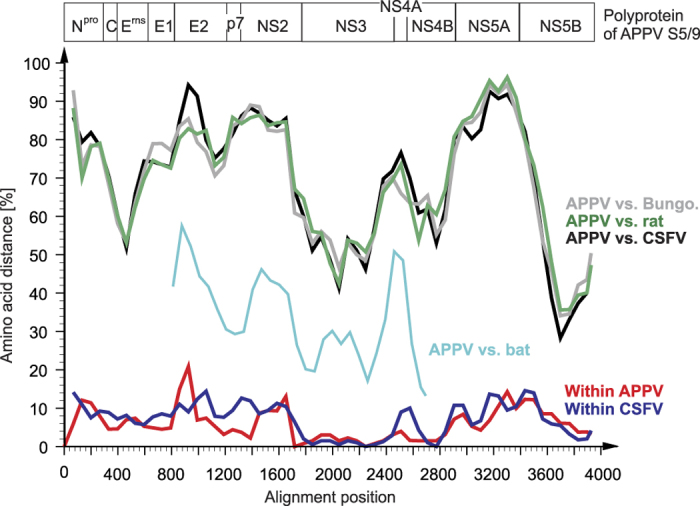
Variability in the polyprotein sequences of atypical porcine pestivirus (APPV) and other pestiviruses. Shown is an amino acid scan performed with the p distance algorithm of the SSE software platform applying a sliding window of 400 and an increment of 200 residues^34^. The novel APPV polyprotein sequence from Germany (S5/9) and the APPV sequence from the USA (GenBank KR011347) were compared to the complete polyprotein sequences of porcine pestivirus Bungowannah (GenBank NC023176), three different CSFV strains, representing genotype 1 (Alfort/187, GenBank X87939), genotype 2 (Alfort-Tuebingen, GenBank J04358) and genotype 3 (94.4/TWN, GenBank AY646427) as well as the complete polyprotein sequence of a pestivirus from a rat (GenBank KJ950914) and a partial pestivirus polyprotein sequence obtained from a bat (GenBank JQ814854). The organization of the APPV S5/9 polyprotein is indicated above the amino acid scan.

**Figure 5:**
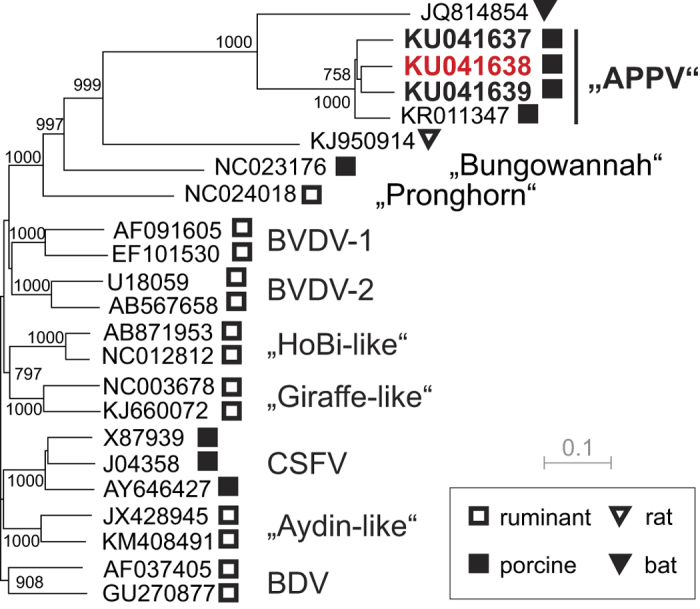
Phylogenetic tree displaying relatedness of atypical porcine pestiviruses (APPV) from three different regions in Germany. Genetic distances were calculated by the Kimura 2-parameter substitution model and phylogenetic analyses by applying the neighbor-joining method as described for CSFV phylogeny^33^. Phylogenetic analysis of partial NS2-3 encoding APPV sequences (1581 nt) from three different regions in Germany (bold) together with respective sequences of established and tentative pestivirus species were analyzed. The GenBank accession number and the corresponding host species (symbols) are indicated for each individual sequence. APPV sequences from Lower Saxony [KU041637] and from Bavaria [KU041639] were obtained from apparently healthy adult pigs. APPV sequence from North Rhine Westphalia [KU041638] was obtained from a congenital tremor diseased piglet (highlighted in red). Bootstrap values were calculated for 1000 iterations. Only significant bootstrap values (≥700) of major nodes are given in the tree. Trees were displayed by dendroscope^38^.

One serum (S5/9), obtained from a sow in Bavaria that revealed to contain highest APPV genome loads, was used to determine the first complete polyprotein coding sequence of European APPV by NGS. The ORF of S5/9 showed 88.2% nucleotide identity to the only so far known APPV sequence from the USA (5) encoding for a polyprotein of the same length (3,635 amino acids). The complete polyprotein sequence of this European APPV revealed an identity of 94.3% to the polyprotein encoded by the American APPV, but only 37.3% identity to the polyprotein of CSFV strain Alfort-Tuebingen^7^. Amino acid variability between the APPV polyprotein sequences from Germany and the USA showed the same range and a very similar pattern of variability as observed among the three established CSFV genotypes (Fig. 4). Similar to the three genotypes of CSFV, the two APPV sequences showed a high degree of conservation in the amino acid composition of nonstructural proteins NS3 and NS5B, but also a significant conservation in NS3 and NS5B when compared to other pestiviruses (Fig. 4). Phylogenetic analyses as well as amino acid scan revealed only a very distant relatedness of APPV to other pestiviruses including CSFV, the atypical porcine pestivirus Bungowannah, a broad spectrum of ruminant pestiviruses, and the recently described pestivirus from Norway rat (Figs 4 and 5). Remarkably, the partial sequence of a pestivirus obtained from a bat (*Rhinolophus affinis*) has an intermediate position between APPV and other pestiviruses, nevertheless showing the typical pattern of conserved and variable regions (Fig. 4). The distances of APPV to the bat pestivirus in the NS2-3 region were 33% on nucleotide level and 26% in the amino acid composition. Analyses of the three partial NS2-3 encoding APPV sequences obtained from Germany displayed similar genetic distances (8.9–10.2%) among each other and to the APPV sequence from the USA (Fig. 5), while the pairwise distances of the respective deduced amino acid sequences were below 2.1% (1.1–2.1%), indicating highly conserved protein functions. In contrast, differences between 51% and 52% were observed on amino acid level to the different CSFV sequences representing the three established CSFV genotypes (Figs 4 and 5).

## Discussion

Pestiviruses are highly variable RNA viruses causing economically relevant diseases in swine, cattle, sheep, and goats. In the last two decades a growing number of novel pestiviruses has been discovered in various domestic and wild ruminant species as well as pigs^4^. The recent identification of highly distinct pestivirus sequences from bats and rats was of particular interest as these discoveries provided the first evidence of pestivirus infections in non-*Artiodactyla* hosts^2^,^3^. An association of these atypical pestiviruses with disease is not known so far. In addition to these findings in wild animals, genomes of another highly distinct pestivirus, tentatively designated “atypical porcine pestivirus” (APPV), were identified in apparently healthy domestic pigs in the USA^6^. The discovery and further characterization of this novel APPV is of particular interest, as other porcine pestiviruses like CSFV and the Australian pestivirus Bungowannah are causative agents of severe diseases in pigs^5^,^8^. An association of APPV with disease remained elusive for many decades. The results of the present study show the association of APPV genomes in the cerebellum and other nervous tissues with the occurrence of CT in newborn piglets. At the time this manuscript was in review, another research group demonstrated an association of APPV and CT in US swine herds by performing an animal experiment with clinical sample material^9^.

CT can be differentiated by the presence (types AI-AV) or absence (type B) of morphological lesions in the brain and spinal cord. Etiologically, CT is associated with infection (AI = CSFV, AII = unknown infectious agent), genetic background (AIII = Landrace; sex-linked recessive), AIV = Saddleback (autosomal recessive; Landrace/Saddleback) and intoxication (AV = metrifonate, trichlorfon)^10^. So far, the etiology of CT type AII remained elusive, although this disease of newborn piglets is known since many decades^11^,^12^. An association of porcine circovirus type 2 (PCV2) with CT type AII was proposed, but several contrary studies could not support this finding^13^,^14^. A recent study reported the presence of astrovirus genomes in the brain of piglets suffering from CT, but RNA loads in the brain appeared to be rather low as a nested-PCR approach was necessary to amplify the viral genomes^15^. Aside from CT caused by CSFV (type AI), a similar clinical presentation is known to occur also in other host species after infection with ruminant pestiviruses like BVDV and BDV (“hairy shaker lambs”). Clinical signs of type AII CT had been experimentally induced in newborn piglets by intramuscular or intrauterine inoculation of pregnant sows with brain suspensions from CT-affected piglets^16^,^17^. In a very recent study, transferability of APPV with serum from a healthy but genome-positive (viremic) sow was demonstrated and inoculation into the fetal amniotic vesicles of a gravid sow resulted in the birth of CT-affected piglets^9^. Experimental infections of pregnant sows with a virus isolate will be required to finally prove that APPV solely can cause CT in newborn piglets and to understand the mechanisms of pathogenesis. However, it was not possible to isolate and propagate the virus so far^6^. Attempts of virus isolation and virus recovery by RNA transfection using various porcine cell lines were not successful and hampered by limited amounts of sample material obtained from the piglets investigated in this study. Therefore, an animal experiment with a virus isolate could not be performed so far. Nevertheless, the detection of high APPV genome loads in the cerebellum, different ganglia and other tissues by qRT-PCR and FISH, including retrospective analyses, together with the recently published data by Arruda *et al*. (2016) strongly suggest that APPV represents a previously unrecognized virus which is associated with the occurrence of CT in piglets.

Intrauterine infections with classical pestiviruses like CSFV, BDV or BVDV are known to induce hypomyelinogenesis and cerebellar hypoplasia^18^,^19^,^20^. Cerebellar hypoplasia is due to a primary infection of the outer granular layer of the cerebellum with consecutive death of the affected neurons^21^. The lack of the outer granular cell layer results in cerebellar hypoplasia with persistent neurological signs since no neurons from the outer granular cell layer can migrate into the inner granular cell layer^21^. Remarkably, in the presented study APPV genomes are most abundantly detected in the cerebellar inner granular cell layer, but not in the outer granular cell layer like observed in infections with classical pestiviruses. This finding may explain the transient clinical signs (recovery of the affected piglets) observed in the present cases since a loss of inner granular cells may be compensated by an immigration of cells from the outer granular cell layer during the first weeks *post natum*. Additionally, the infection of spinal ganglia may contribute to the observed clinical signs in infected piglets. However, since no inflammation was detected within the central and peripheral nervous system of piglets with CT the mechanism of virus clearance remains elusive so far. Distribution of virus within herds may occur via the orofecal route since a significant amount of APPV genome was present in salivary glands, duodenum, pancreas and colon. Future studies will address the transmission routes of this novel virus.

*Postmortem* investigation of CT type II diseased piglets revealed varying degrees of hypomyelination of brain and spinal cord^22^. In the present study, a mild reduction of myelin in the spinal cord was observed in four out of six affected piglets. As two APPV positive piglets showed no convincing hypomyelination despite shivering, this morphologic finding probably results from a transiently delayed myelination. A transient course of infection and subsequent completion of myelogenesis is likely to occur in the piglets as the majority of affected piglets recover after some time (2–3 weeks). In addition, the lack of APPV genomes observed in sows with affected litter gives strong evidence for a transient and obviously clinically inapparent infection during gestation. This is in line with a previous study reporting that an infection of adult sows with clinical sample material did not result in a clinical manifestation except for the production of CT-affected piglets showing varying degrees of hypomyelination of brain and spinal cord^22^. Screening for APPV genomes in a larger number of gilts from the affected farms will help to identify acute infections during gestation and will provide information with respect to the clinical signs in sows under field conditions. In addition, serial bleeding of affected piglets will be performed in the future to answer the question whether abrogation of clinical signs coincidence with clearance of viremia or whether recovered piglets remain persistently infected. First results show that some of the CT affected piglets still have high genome loads (Cq values 21–24) in the serum at the age of 31 days, albeit showing eased clinical signs. Based on the knowledge of other pestiviruses it can be speculated that chronically or persistently infected, but clinically healthy pigs shedding the virus are the source of infection for serologically naïve sows, which are newly introduced in a herd with these viremic animals and subsequently experience a transient infection with significant consequences for the unborn piglets in case of pregnancy. This hypothesis – which is supported by the current knowledge in CT type AII^23^ – is further strengthened by the epidemiological investigation of the APPV associated outbreak of CT described here. A sero-negative APPV status of the farm delivering the gilts to the CT-affected farms (with putatively high seroprevalence of APPV specific antibodies) would strongly support this hypothesis. A novel serological assay for APPV is in development to address the epidemiology and the seroprevalence of APPV.

Despite all differences of APPV to classical pestiviruses, the APPV genomes from Europe and the USA encode for proteins being unique for members of the genus *Pestivirus*, namely the N-terminal protease N^pro^ and the secreted glycoprotein E^rns^ containing also the conserved motif required for RNAse activity. The different APPV sequences characterized in this study revealed significant differences to the reference sequence from the USA, but also between the APPVs originating from three different regions in Germany. This finding points towards geographically isolated virus populations in Germany, which must have evolved over a longer period of time and are epidemiologically not linked with each other. In addition, the observed genetic variability and the distribution of variable and conserved regions in the polyprotein of the characterized APPV sequences are comparable to the variability among different CSFV genotypes (Fig. 4). Considering the high variability of the characterized APPV genomes and their estimated prevalence in domestic pigs, it appears likely that infections with these newly discovered pestiviruses frequently occur, but without knowledge of its association with CT in newborn piglets remained clinically unrecognized in the past.

In the presented study, we identified APPV genomes in the cerebellum and ganglia of new-born piglets suffering from CT type AII. This strongly suggests that APPV infection contributes to the induction of CT in piglets. In addition, APPV sequences can be detected in clinically healthy adult pigs. Future studies will address the biology of this atypical pestivirus and help to reduce losses in pig production by tailored herd management and prevention strategies.

## Materials and Methods

### Sample material

369 serum samples from clinically unsuspicious sows and fattening pigs originating from Bavaria (20 farms, 200 sera), Lower Saxony and Schleswig-Holstein (8 farms, 158 sera) were obtained in the framework of veterinary microbiological diagnostics or the Salmonella monitoring program in accordance to German legislation (SchwSalmoV §2) and residual volumes of these samples were provided for use in the present study. Therefore no ethical approval was required for the use of these samples. Eleven samples were residual volumes obtained from a previous experimental study conducted in Schleswig-Holstein, which was notified and approved by the local authorities (Ministry of Energy, Agriculture, the Environment and Rural Areas, Schleswig-Holstein: reference number V244-7224.121.9-34). Samples and piglets from the CT-affected farms were sent to the University of Veterinary Medicine, Hannover, for diagnostic reasons, especially for the exclusion of CSF, which is in accordance to German legislation (SchHaltHygV §8) and does not require further approval by the authorities. None of the animals included in this study was infected experimentally.

### Post mortem examination and sample collection

Serum, cerebrospinal fluid and tissue samples from different organs were taken from CT-affected (n = 6) and unaffected (n = 2) piglets of different litters at the age of two days. For histology, all tissues were routinely processed in paraffin wax, cut at 2 μm thickness, and stained with hematoxylin and eosin. In addition, cross-sections of spinal cord and cerebellum were stained with Luxol fast blue for the investigation of myelination as previously described^24^,^25^.

### RNA isolation

RNA from liquids (serum, cerebrospinal fluid) was prepared with the ViralAmp Kit (Qiagen, Hilden) and RNA from formalin-fixed archived cerebellum samples was extracted with the RNeasy FFPE kit according to the recommendations of the manufacturer (Qiagen, Hilden). RNA from tissues was isolated by phenol-chloroform precipitation or with the Nucleospin RNA kit (Macherey-Nagel, Düren).

### Reverse transcription PCR (RT-PCR)

The presence of CSFV and genomes of other established pestiviruses was excluded by applying the accredited methods of the EU and OIE Reference Laboratory for CSF, Hannover, using primers described previously^26^,^27^. Based on the only available APPV sequence and the sequences of atypical pestiviruses from bat (*Rhinolophus affinis*) and rat (*Rattus norvegicus*), primers targeting conserved regions in the NS3 (PCR 1) and the NS4B (PCR 2) encoding regions were designed for APPV screening (Table 2). These APPV screening PCRs were performed using the QuantiTect SYBR-Green kit (Qiagen, Hilden) with subsequent visualization of the PCR products by agarose gel electrophoresis. Quantitative TaqMan-PCR was performed based on the established real-time PCR protocol of the EURL with the QuantiTect Probe RT-PCR kit (Qiagen, Hilden) containing 1.5 pmol of each primer used in APPV-specific screening PCR 1 and 0.25 pmol probe (specific for the APPV sequence obtained from CT-affected piglet no. 59) per 20 μl mastermix. For all PCR reactions five microliters of RNA were added to the mastermix and amplification was performed in a one-step PCR reaction with 50 °C, 30 min; 95 °C, 15 min and 40 cycles comprising 95 °C, 30 sec; 56 °C, 30 sec; 72 °C, 30 sec.

### APPV fluorescent *in-situ* hybridization (FISH)

A broad spectrum of formalin-fixed, paraffin-embedded tissues from the piglet (no. 59) with the highest amount of APPV genome, cerebellum from two non-shivering piglets as well as cerebellum from two shivering piglets necropsied in 2007 were analyzed using FISH, as previously described^28^, with a probe targeting a fragment of the NS3 encoding sequence of APPV (GenBank KU041638) and a probe specific for porcine ubiquitin as positive control with the following modifications: Pretreatment of tissue sections included boiling (85–90 °C) in pretreatment solution (Affymetrix-Panomics, Santa Clara, CA) for 20 minutes, followed by protease QF (Affymetrix-Panomics) digestion for 10 minutes at 40 °C. Hybridization, pre-amplification, amplification, and detection were performed according to the manufacturers’ instructions. Images were acquired with a color video camera (DP72, 12.8 megapixel CCD; Olympus, Hamburg, Germany) mounted on an IX50 microscope (Olympus) using the cellF Software (version 3.3; Olympus, Hamburg, Germany).

### Determination of nucleotide sequences

The complete polyprotein encoding sequence of one APPV-positive porcine serum sample from Bavaria (sample S5/9) was determined by next generation sequencing on an Illumina HiSeq (2500 2 × 150 bp paired end run, sequencing depth: 6 Mio reads) as recently described^29^,^30^. The complete genome was assembled using the IDBA-UD algorithm with a minimum coverage of 36.8 31.

For amplification and determination of partial NS2-3 encoding sequences complementary DNA was transcribed using Superscript II reverse transcriptase and random hexamers. Amplification was performed in three different PCRs using the AccuPrime polymerase (LifeTechnologies, Darmstadt) and primers indicated in Table 2. The nucleotide sequences (1581 nucleotides) of the obtained PCR products were determined by conventional Sanger sequencing (LGC genomics, Berlin).

### Sequence analyses

To genetically characterize the identified APPV, the partial NS2-3 encoding sequence (1581 nucleotides) obtained from a CT-affected piglet in 2015 (North Rhine Westphalia) was compared to APPV sequences obtained from sera of one clinically unsuspicious fattening pig from Lower Saxony and one unsuspicious sow originating from Bavaria. Multiple sequence alignments were generated with ClustalW of the Multiple Sequence Comparison by Log-Expectation (MUSCLE) tool provided by EMBL-EBI^32^. Genetic distances were calculated with the Kimura 2-parameter substitution model and phylogenetic analysis was performed by the Neighbour-joining method as previously reported for pestiviruses^4^,^33^. Amino acid scan of polyprotein sequences was performed with the sequence distance calculation tool of the SSE software platform using the p distance method and a sliding sequence window of 400 residues with 200 residues increment^34^.

### Virus isolation

Attempts of virus isolation were performed with tissue homogenate (undiluted and a 1:10 dilution) of the diseased and highly APPV genome positive piglet #59 (*Arcus palatoglossus*; Cq-value 17) on cell lines susceptible for established pestiviruses (PK-15, SK6 and STE cells) according to the accredited protocol of the EU and OIE Reference Laboratory for CSF. In addition, the tissue homogenate as well as APPV genome positive pig sera from Bavaria and Lower Saxony were incubated on porcine lymphoma cells 38A1D and porcine endothelial cells PEDSV.15 35,36. RNA preparations of the tissue homogenate and of a viremic pig serum (S5/9, Bavaria) were used to transfect SK6 cells as described previously^37^. At least three serial blind passages were performed followed by qRT-PCR screening of cells and supernatants for the presence of APPV genomes.

## Additional Information

**Accession codes**: The obtained consensus sequences were deposited in GenBank (KU041637 - KU041639).

**How to cite this article**: Postel, A. *et al*. Presence of atypical porcine pestivirus (APPV) genomes in newborn piglets correlates with congenital tremor. *Sci. Rep.* **6**, 27735; doi: 10.1038/srep27735 (2016).

## Figures and Tables

**Table 1:** Association of congenital tremor with atypical porcine pestivirus (APPV) genome detection.

Piglet [ID]	Congenital tremor	APPV genome detection in qRT-PCR [Cq values]			
		Serum	CSF^a^	CNS pool^b^	Cere bellum
51	−	−	−	−	−
52	+	29.7	28.1	27.0	25.8
53	−	−	−	−	−
54	+	29.3	27.0	26.6	27.5
55	+	29.9	27.5	30.6	21.9
56	+	27.4	27.2	28.0	24.8
57	+	27.3	28.4	29.8	24.9
59	+	27.4	26.0	22.3	21.0

^a^Cerebrospinal fluid.

^b^Central nervous system comprising cerebrum, cerebellum, spinal cord.

**Table 2:** Atypical porcine pestivirus (APPV)-specific primers and probes used in the study.

Primer/Probe	Sequence (5′-3′)	Target	Purpose
APPV_5587-fw	CAGAGRAAAGGKCGAGTGGG	NS3	PCR 1, qRT-PCR
APPV_5703-rev	ACCATAYTCTTGGGCCTGSAG		PCR 1, qRT-PCR, sequencing PCR A
APPV_CT-59 probe	[6FAM] ACTACTATCCTTCGGGGGTAGTACCGA [BHQ1]		qRT-PCR
APPV_6869-fw	CTTTCATGGARTCWGGCGGTG	NS4B	PCR 2
APPV_6950-rev	AGACTCCTRTTTTCTGCATGTT		PCR 2
APPV_5087-fw	GAAAGTGTCTGCCGCTTCATG	NS3	sequencing PCR A
APPV_4186-fw	GTGCGGCCTCCCAACTGTAG	NS2	sequencing PCR B
APPV_4273-fw	TGGGGACCTCACCAGTGATG	NS2	sequencing PCR C
APPV_5169-rev	ACGTCACCCTCTTTCCGCTC	NS3	sequencing PCR B/C
